# Enterovirus D68–Associated Acute Flaccid Myelitis, United States, 2020

**DOI:** 10.3201/eid2610.201630

**Published:** 2020-10

**Authors:** Sarah Kidd, Adriana S. Lopez, Jennifer L. Konopka-Anstadt, W. Allan Nix, Janell A. Routh, M. Steven Oberste

**Affiliations:** Centers for Disease Control and Prevention, Atlanta, Georgia, USA

**Keywords:** acute flaccid myelitis, enterovirus infections, central nervous system viral diseases, viruses, United States, EV-D68

## Abstract

Acute flaccid myelitis (AFM) is a serious neurologic condition that causes limb weakness or paralysis in previously healthy children. Since clusters of cases were first reported in 2014, nationwide surveillance has demonstrated sharp increases in AFM cases in the United States every 2 years, most occurring during late summer and early fall. Given this current biennial pattern, another peak AFM season is expected during fall 2020 in the United States. Scientific understanding of the etiology and the factors driving the biennial increases in AFM has advanced rapidly in the past few years, although areas of uncertainty remain. The Centers for Disease Control and Prevention and AFM partners are focused on answering key questions about AFM epidemiology and mechanisms of disease. This article summarizes the current understanding of AFM etiology and outlines priorities for surveillance and research as we prepare for a likely surge in cases in 2020.

AFM is a syndrome characterized by the acute onset of flaccid limb weakness and lesions in the gray matter of the spinal cord visible on magnetic resonance imaging; the lesions represent damage to the lower motor neurons in the anterior horns. This feature distinguishes AFM from other disorders associated with acute flaccid limb weakness or paralysis, such as disorders of peripheral nerves (e.g., Guillain-Barré syndrome) or neuromuscular transmission (e.g., myasthenia gravis or botulism). AFM can be caused by multiple infectious and noninfectious etiologies. Poliovirus, nonpolio enteroviruses, and flaviviruses are all known to cause AFM in a subset of persons who are infected (*1*–*4*). In addition, noninfectious etiologies such as neuroinflammatory conditions or spinal vascular disease can result in a clinical and radiographic picture that overlaps with that of AFM caused by infection (*5*–*7*). Although the clinical severity of AFM is variable, it can progress rapidly and lead to respiratory compromise requiring mechanical ventilation (*8*–*10*). At present, no proven treatments for AFM have been identified. Although some patients recover function, AFM is frequently associated with long-term neurologic deficits and impairment (*11*,*12*).

Historically, the United States conducted surveillance for acute flaccid paralysis (AFP) associated with poliovirus infection. Poliomyelitis and detection of poliovirus infection remain nationally notifiable conditions in the United States. After the introduction of poliovirus vaccine and subsequent elimination of indigenous poliovirus in the United States in 1979, AFP epidemics in the United States also appeared to have been eliminated. However, in 2014 a cluster of 9 pediatric AFM cases of unknown etiology was observed in Colorado, and 23 similar cases with onset during 2012–2014 were reported from California (*13*,*14*). In response, the Centers for Disease Control and Prevention (CDC) called for additional case reports and a total of 120 cases were confirmed nationally in 2014; all tested stool specimens were negative for poliovirus, and no cases were epidemiologically linked to poliovirus. Subsequently, national surveillance for the syndrome of AFM was initiated using a standardized case definition that distinguished AFM from AFP associated with poliovirus (*15*). Since that time, nationwide outbreaks have occurred in 2016 and 2018 (*10*) (Figure). The epidemic curve demonstrates the seasonal periodicity of AFM; more than two thirds of peak-year cases are in patients with illness onset occurring August–October. In 2018, the most recent peak year, a total of 238 confirmed cases were reported from 42 states to CDC (https://www.cdc.gov/acute-flaccid-myelitis/cases-in-us.html). Although national case reporting did not start until late 2014, additional data indicate that this epidemiologic pattern in the United States is new. A study that retrospectively searched for cases among magnetic resonance imaging results and electronic medical records at 5 large pediatric medical centers found low numbers of AFM cases before 2014 but an increase in cases in 2014 (*16*), suggesting a new or changing etiology of AFM.

**Figure:**
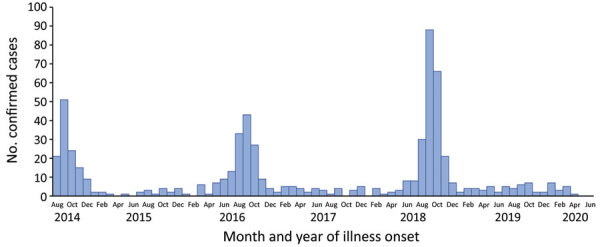
Number of confirmed cases of acute flaccid myelitis reported to the Centers for Disease Control and Prevention, United States, August 1, 2014–June 30, 2020. Data as of July 31, 2020.

Identifying the cause of the recent change in AFM epidemiology and understanding how it leads to this specific neurologic disease are critical for developing effective interventions to prevent and treat AFM. The clinical manifestations and epidemiology of confirmed AFM cases strongly suggests an infectious etiology, probably viral. Most AFM cases are in patients with prodromal symptoms consistent with a viral illness before onset of limb weakness, and AFM patients in peak years are significantly more likely to have prodromal respiratory illness or fever than those in nonpeak years (*9*,*10*,*17*). In addition, the increase in AFM noted in 2014 coincided with an unusual increase in severe respiratory illness caused by enterovirus D68 (EV-D68) in the United States; 11 (20%) of 56 AFM patients whose respiratory specimens were tested at CDC in 2014 were positive for EV-D68 (*8*,*18*). However, because virus detection in the cerebrospinal fluid (CSF) or other sterile site specimens of AFM patients is rare and not consistent by type, definitively establishing a classic causal relationship between viral infection and AFM is challenging (*8*–*10*).

Nevertheless, data accumulated over the last 5 years indicate that enteroviruses, and more specifically EV-D68, are a major factor of the new AFM epidemiology (*19*). Although examination of CSF from AFM patients rarely yields a pathogen, 2 recent studies used novel techniques to document the presence of enterovirus-binding antibodies in the CSF of AFM patients (*20*,*21*). In both studies, AFM patients were more likely than non-AFM controls to have enterovirus-specific antibodies identified in their CSF. Although neither study documented the presence of IgM in CSF, which would indicate intrathecal synthesis of enterovirus antibodies and be considered nearly definitive proof of central nervous system (CNS) infection, and controls in these studies were imperfectly matched to cases for age, year, and season, these data provide evidence for a causal relationship between enteroviruses and AFM. A temporal association exists between EV-D68 circulation and increases in AFM (*22*,*23*), and a case-control study has demonstrated that children with AFM were more likely to be infected with EV-D68 than children tested for respiratory illness in the outpatient setting during the same period (*24*). As with many other nonpolio enteroviruses, EV-D68 can cause paralysis in experimental mouse models (*25*–*28*), thus establishing a plausible, but not unique, causal relationship between EV-D68 infection and AFM. Since 2014, enteroviruses and rhinoviruses (EV/RVs) have been the most common pathogens detected in clinical specimens from AFM patients, primarily among respiratory specimens, and EV-D68 is the single most common virus detected (*8*–*10*). In 2016 and 2018, 38% of all AFM patients with >1 clinical specimen tested at CDC were positive for EV/RV (*17*). EV-D68 was detected in 21%, enterovirus A71 (EV-A71) in 5%, and various other EV/RV were detected in 12%. Yield of EV/RV and EV-D68 testing among AFM patients is dependent on timing and type of specimen collection and is higher among respiratory specimens collected within 5–7 days of respiratory or febrile illness onset (*8*,*9*). Of note, EV-D68 was not detected in specimens from any patients with onset during the nonpeak years of 2015 and 2017, suggesting that EV-D68 is playing a role in the increases in AFM cases every 2 years but plays less of a role during nonpeak years (*17*). In contrast, most EV-A71-positive specimens were associated with a cluster of AFM cases in Colorado in 2018 (*29*). Together, these epidemiologic and laboratory data indicate that enteroviruses, and EV-D68 in particular, are the main etiology underlying the observed increases in AFM during 2014, 2016, and 2018. However, the EV-A71 cluster in 2018 is a reminder that even in peak years, more than 1 enterovirus can cause AFM, warranting continued clinical and laboratory surveillance to understand the full etiologic spectrum of AFM and the mechanism (or mechanisms) of AFM pathogenesis.

Despite the evidence supporting EV-D68 as the primary cause of AFM during peak years, areas of uncertainty remain. The reason that diagnostic testing rarely detects EV-D68 or other pathogens in the CSF of AFM patients, even when broad metagenomic methods are used (*8*,*20*,*21*), is unclear. The virus might be rapidly neutralized and cleared from the CNS. Alternatively, the virus might be present in neural tissue but not released into the CSF. Historically, poliovirus was also rarely identified by cell culture in the CSF of patients with paralytic poliomyelitis but was commonly identified in stool (*30*). Modern molecular methods probably have greater sensitivity than cell culture, but CSF has rarely been collected from AFP patients with poliovirus infection during the era of molecular testing era, so making a direct sensitivity comparison is difficult. Timing of specimen collection probably explains at least part of the low virus detection in sterile site clinical specimens in AFM. In 2018, the median interval from onset of limb weakness to specimen collection was 2 days for CSF (range 0–31 days, interquartile range 1–4 days), and 3 days for respiratory specimens (range 0–35 days, interquartile range 2–6 days) (*10*). Most patients with confirmed AFM report the onset of prodromal fever, upper respiratory illness, or both a median of 5 days before onset of limb weakness, meaning that up to half of specimen collection is occurring >7 days after initial illness onset (*10*). Because respiratory specimens collected earlier in the course of AFM have had higher pathogen yield (*8*,*9*), these data underscore the importance of early recognition of symptoms and timely specimen collection to improve etiologic studies of AFM.

Another area of uncertainty is how to reconcile the apparent relationship between EV-D68 and AFM with serologic data indicating that EV-D68 is and has been a common infection for decades. Studies from multiple countries demonstrate a high prevalence of serum antibodies and nearly universal exposure to EV-D68 before adulthood, with most children developing antibodies by 2–5 years of age (*31*–*34*). If EV-D68 is the main cause of recent increases in AFM, why does an apparently ubiquitous infection lead to neurologic complications in only a small proportion of infected persons? Determining genetic and other risk factors that explain why some persons might be more likely to have onset of AFM as a consequence of EV-D68 infection is an active area of AFM research. Further, given serologic evidence that EV-D68 infection was common before 2014, what changed to trigger the observed increase in AFM in 2014 despite a largely serologically positive population, and why has the number of AFM cases continued to peak every 2 years in the United States? Although the reasons for the biennial pattern of AFM are not yet understood, it is notable that other viruses have circulated in biennial patterns, especially when population immunity is high and when the number of unexposed susceptible persons is insufficient to sustain transmission in the population every year (*35*,*36*). As infants are born each year, the number of unexposed susceptible infants and young children might accumulate and reach a threshold that sustains increased transmission of the virus approximately every 2 years. In that scenario, one would expect most of the cases to be among children born after the previous peak (i.e., those <2 years of age). However, AFM patients have a median age of 5 years and have a relatively wide age range, which argues against this explanation for observed AFM trends.

Moreover, if EV-D68 is the main cause of recent increases in AFM, the occurrence of disease among a population with apparently high prevalence of serum neutralizing antibody raises questions about whether serum antibodies are protective against EV-D68 infection. Current serologic assays might be flawed, or children with AFM might represent the small percentage of children who somehow escaped primary infections as infants or young children. Alternatively, as in the case of most respiratory viruses, serum antibodies resulting from prior exposure to EV-D68 might not be sufficient to prevent re-infection. Mucosal antibodies or other components of immunity might be more closely correlated with protection from disease.

Furthermore, an important consideration is whether serum antibodies or prior homologous or heterologous viral exposure could increase the risk for AFM through a mechanism such as the antibody-dependent enhancement that has been observed in dengue virus infection (*37*,*38*) and has been hypothesized for infection with coxsackie B viruses and EV-A71 on the basis of data from cell culture and animal models (*39*–*41*). Under this hypothesis, antibodies developed during an initial viral infection do not neutralize the virus when a person is later re-infected. Instead, the primary antibodies developed during the initial infection facilitate infection of monocytes through Fc receptors during the subsequent infection, resulting in increased viral replication and higher risk for severe disease. At present, the data are insufficient on the relationship between serostatus or prior infection with EV-D68 and risk for re-infection. Further research on these issues related to immunity will be critical for understanding the pathogenesis of AFM and for development of effective treatment and prevention strategies.

The most critical unknown is the underlying mechanism by which EV-D68 and other enteroviruses cause AFM. Specifically, the extent of damage caused by direct viral invasion and the role of the subsequent inflammatory and immune response, if any, are unclear. Recent studies have demonstrated that EV-D68 can enter neurons, replicate, and cause neurotoxic infection in cell culture and animal models (*25*,*42*,*43*). At present, no direct evidence exists for autoimmune-mediated neuronal damage in AFM. The relatively short interval (median 5 days) (*10*) between onset of prodromal febrile respiratory illness and onset of limb weakness suggests spinal cord neuronal damage is caused by direct viral injury and possibly the immediate inflammatory response, as opposed to an antibody-mediated response with autoantibodies directed at host antigens, which probably would require more time to develop. However, given that the initial infection probably precedes the prodrome by several days, involvement of antibody-mediated mechanisms cannot be excluded. Differentiating clinical case characteristics in peak AFM years compared with nonpeak years might provide clues about viral pathogenesis. For instance, AFM patients in peak years were more likely to have upper limb involvement compared with patients in nonpeak years (*17*). In contrast, a predominance of lower limb involvement is observed in patients with paralytic poliovirus infection (*30*). Viruses that infect and replicate in the respiratory tract, such as EV-D68, might be more likely to invade and affect the cervical portion of the spinal cord, resulting in upper extremity involvement, whereas viruses with fecal-oral spread that replicate in the gastrointestinal tract, such as poliovirus, might be more likely to invade the lower portions of the cord and result in lower extremity paralysis. Additional research to clarify how EV-D68 travels from the presumed site of entry to the CNS and the mechanism by which it causes injury to neurons will also be critical to identifying potential targets and interventions to treat and prevent AFM.

Another rise in AFM cases is anticipated in 2020 and, to address existing gaps in knowledge, CDC and other partners working on AFM have prioritized several surveillance and research activities in preparation for the expected increase. At CDC, numerous activities to strengthen AFM surveillance have been implemented. First, in addition to CDC’s ongoing funding of 60 jurisdictions for AFM surveillance, CDC provided additional funding to 10 jurisdictions to conduct special projects aimed at improving AFM case ascertainment and reporting through active surveillance, outreach, and education activities. Second, CDC is funding a pilot study to improve case finding and decrease lag time in reporting to health departments. Third, CDC has implemented enhanced prospective and retrospective AFM surveillance through the New Vaccine Surveillance Network (NVSN). NVSN conducts systematic, active, population-based viral surveillance and testing among children with gastrointestinal and respiratory infections at 7 pediatric hospitals throughout the country. Seasonality of enteroviruses varies by geography in the United States. To date, the relatively small numbers of AFM cases and the lack of systematic virologic surveillance has hindered the ability to determine the correlation between enterovirus and AFM epidemiology. These viral and AFM surveillance data from NVSN will be key to documenting and understanding patterns of enterovirus circulation in the United States, correlating trends in virus circulation with trends in AFM, and understanding the apparent change in AFM epidemiology since 2014.

CDC, the National Institutes of Health (NIH), and academic partners are also engaged in various laboratory studies to better characterize AFM etiology and understand pathogenesis. One theory for the apparent sudden emergence of EV-D68 and AFM in 2014 is a change in the virus, resulting in alterations in tissue tropism, neurovirulence, or other key pathogenic properties, and some data support this theory (*42*). However, studies have not identified a clear viral genetic signal that consistently correlates with neurologic disease, and data from other investigators indicate that neurotropism is not a recently acquired phenotype (*44*). Studies of EV-D68 tropism and replication in cell culture models, including those of neural or respiratory origin, might identify more subtle evolutionary changes that influence pathogenesis. Given that identification of a pathogen in the CSF of AFM cases has proven elusive, the CDC AFM laboratory has expanded its focus from direct pathogen detection to identification of indirect evidence for infection and possible immune correlates of disease. CDC will continue to investigate the relationship between EV-D68 and AFM through serologic assays (i.e., neutralization and IgM assays) and detection of virus-specific B cells. Investigations have also been broadened by examining soluble and cell-associated markers of immune system activation, particularly in the CNS, and other immune-mediated mechanisms. Much of this work could inform the development of new diagnostic assays that might enhance our ability to define AFM cases. The CDC AFM laboratory is also conducting a national, population-based EV-D68 serosurvey, testing samples from 1999–2018, which will advance our knowledge about the prevalence of EV-D68 antibodies in the United States in various birth cohorts over time and help to elucidate the relationship between serum EV-D68 antibodies and AFM.

Additional priority activities to further understanding of AFM pathophysiology include a collaboration between NIH, academic partners, and CDC on the NIH-funded AFM natural history study (https://www.uab.edu/medicine/peds/casg/current-studies/acute-flaccid-myelitis-study), which will follow AFM patients longitudinally and collect specimens as well as detailed information about their course of illness, treatment, and potential risk factors for illness and severity of disease. CDC is also implementing a protocol that will facilitate the collection of specimens from AFM patients who are not enrolled in the natural history study. Specimens from the NIH natural history study and the CDC protocol will be stored in a common biorepository to be used for future research. This biorepository will be a resource for researchers who are investigating the AFM pathogenesis and potential biologic and genetic risk factors associated with this devastating illness.

The ultimate goal of these activities is to acquire critical knowledge that will lead to the development of diagnostic tests, treatments, and prevention of AFM and its sequelae. Currently, the response to AFM is unlike most other public health responses, such as for polio, where proven prevention activities to slow or stop an outbreak exist. Nevertheless, clinical and public health actions can have an impact on patient care. Therefore, a key portion of CDC preparedness activities focus on communications that increase clinician and public awareness of AFM, leading to more rapid identification and appropriate management of patients. To aid in case recognition and management, CDC has been conducting market research to improve communication strategies with clinicians, particularly among emergency and urgent care clinicians who are on the frontlines and often are the first to see patients with limb weakness. CDC has also been updating its AFM web content, including clinical guidance for managing AFM and educational materials for 2020. Together with partnerships that include the AFM Working Group and the Siegel Rare Neuroimmune Association, CDC has facilitated linkages for providers to receive expert AFM consultation through the Siegel Rare Neuroimmune Association provider consultation portal (https://wearesrna.org/living-with-myelitis/resources/afm-physician-support-portal). CDC is also continuing to engage with the AFM parent network, who continue to be key partners in efforts to improve public awareness of AFM ahead of the possible 2020 surge.

The effect of the current coronavirus disease pandemic on AFM epidemiology is difficult to predict. Physical distancing measures, if sustained, could potentially decrease community enterovirus transmission and dampen or delay the expected increase in AFM cases in 2020. Certainly, the pandemic’s impact on the health system will pose additional challenges to addressing AFM in 2020. Because AFM remains a high priority, CDC and partners will continue to promote critical, targeted outreach and education for clinicians and parents to have a high degree of suspicion for AFM during this period. By raising clinician awareness, providing clinical tools like the provider consultation portal, and conducting robust communication activities to keep the public informed, it is hoped that patients can be recognized earlier, hospitalized rapidly, and receive appropriate management that might positively affect their illness outcome.

Recent data indicate that EV-D68 is the probable cause of the recent increases in AFM cases. However, other viruses, including other enteroviruses, are certainly contributing to disease, and etiologies of AFM might shift over time. Ongoing surveillance and investigation are needed to monitor and detect potential shifts in etiology. In addition, many unanswered questions remain about how EV-D68 causes AFM and why EV-D68–associated AFM appears to have emerged in or shortly before 2014. As we anticipate another increase in AFM cases in the United States during the second half of 2020, national partnerships built since 2018 are providing mechanisms to study the epidemiology, etiology, and pathogenesis of AFM. Through collaborations between federal agencies, academic partners, and parent organizations, opportunities to better understand the natural history of AFM and collect specimens for critical research on pathogenesis are now available. CDC’s case-based and virologic surveillance will continue to generate critical epidemiologic, etiologic, and pathogenesis data to support treatment and vaccine development. Together, this robust AFM network can create a framework for preventing AFM and its devastating outcomes.
